# Design, implementation, and evaluation of a programmatic assessment model in an emergency medicine residency: a mixed-methods study

**DOI:** 10.3389/fmed.2026.1881471

**Published:** 2026-09-03

**Authors:** İbrahim Ulaş Özturan, Dilek Kitapcioglu, Prattama Santoso Utomo, Cansu Alyesil Ozturan, Nurettin Özgür Doğan, Elif Yaka, Serkan Yılmaz, Murat Pekdemir, Levent Altıntaş

**Affiliations:** 1 Department of Medical Education, Acibadem University, Istanbul, Türkiye; 2 Department of Emergency Medicine, Kocaeli University Faculty of Medicine, Kocaeli, Türkiye; 3 International FAIMER Institute, Foundation for Advancement of International Medical Education and Research, Philadelphia, PA, United States; 4 Department of Medical Education and Bioethics, Faculty of Medicine, Public Health and Nursing, Universitas Gadjah Mada, Yogyakarta, Indonesia; 5 Department of Emergency Medicine, Kocaeli City Hospital, Ministry of Health, Kocaeli, Türkiye

**Keywords:** competency-based medical education, emergency medicine, mixed-methods study, postgraduate medical education, programmatic assessment

## Abstract

**Background:**

Programmatic assessment (PA) is widely endorsed in postgraduate medical education, but empirical accounts of real-world implementation in emergency medicine (EM) remain limited and are concentrated in North American settings. We piloted and evaluated the design, implementation, and outcomes of a PA model in an EM residency at a high-volume tertiary care center in Türkiye.

**Methods:**

A mixed-methods study was conducted over an eight-month pilot (November 2024–June 2025). Program design and evaluation followed a Theory of Change framework applied across three sequential phases: a structured gap analysis informed by external medical-education experts, competency-based blueprint development across six domains, and pilot implementation in a small core-faculty and resident cohort. Outcomes included feasibility (assessment volume and participation), cohort-level educational impact (longitudinal performance via paired-sample comparisons), and acceptability (as measured by an end-of-pilot satisfaction survey and qualitative feedback).

**Results:**

A total of 3,107 assessment data points were documented across all six competency domains. Among 16 residents with paired baseline and follow-up data, cohort-level relative performance increased significantly in five of six domains. Evidence-Based Medicine and Lifelong Learning did not change significantly. The survey response rate was 89.5%, and 82.4% of respondents endorsed continuing the program. Acceptability data converged across streams: residents simultaneously valued the multisource perspective and voiced concerns about feedback culture, with the burden of form completion as the dominant practical concern.

**Conclusions:**

Implementation was feasible, accompanied by positive change in cohort-level relative performance, and was broadly acceptable. The most informative finding was a tension between the system's formative design intent and how some residents experienced it, which directly informs next-cycle priorities focused on feedback culture, technology integration, and sustainability.

## Introduction

Assessment in postgraduate medical education serves not only to certify competence but also to guide learning and professional development. Traditional assessment models in residency training, however, frequently rely on fragmented, episodic evaluations that fail to provide continuous longitudinal insight into competency development.

Programmatic assessment (PA) has emerged as a theoretical and practical response to these limitations (1–3). Rather than treating assessments as isolated events, PA conceptualizes assessment as a coordinated system in which multiple low-stakes data points are aggregated over time to inform both formative feedback and high-stakes decision-making (2). Core principles include triangulation across tools, longitudinal data collection, and narrative-rich feedback to support developmental progression (3, 4). Despite strong conceptual support, translating PA theory into sustainable practice within busy clinical training environments remains challenging (5–8).

Empirical work on PA implementation in postgraduate medical education has accumulated over the past decade across emergency medicine, family medicine, internal medicine, anesthesiology, and surgery (6–19). This literature documents both the feasibility of data accrual and recurring concerns regarding workload, rater behavior, integration of direct observation, and the formative–summative balance. Reports remain concentrated in North American programs, almost exclusively so within emergency medicine. Settings with different workforce structures, faculty-to-resident ratios, and assessment cultures are largely absent from the published literature (5).

In Türkiye, specialty residency training — including EM — is structured under national competency frameworks but has historically relied on summative knowledge testing as the primary mode of assessment. This contextual difference, together with workforce and faculty-to-resident structures distinct from those of the more developed and standardized systems that dominate the published PA literature, offers a useful test of the portability of programmatic assessment. We therefore set out to design, implement, and evaluate a pilot programmatic assessment model in this setting, addressing the local gap between fragmented existing assessment practices and national competency standards while generating transferable evidence for programs operating under similar constraints.

## Methods

### Study design and setting

**Figure 1:**
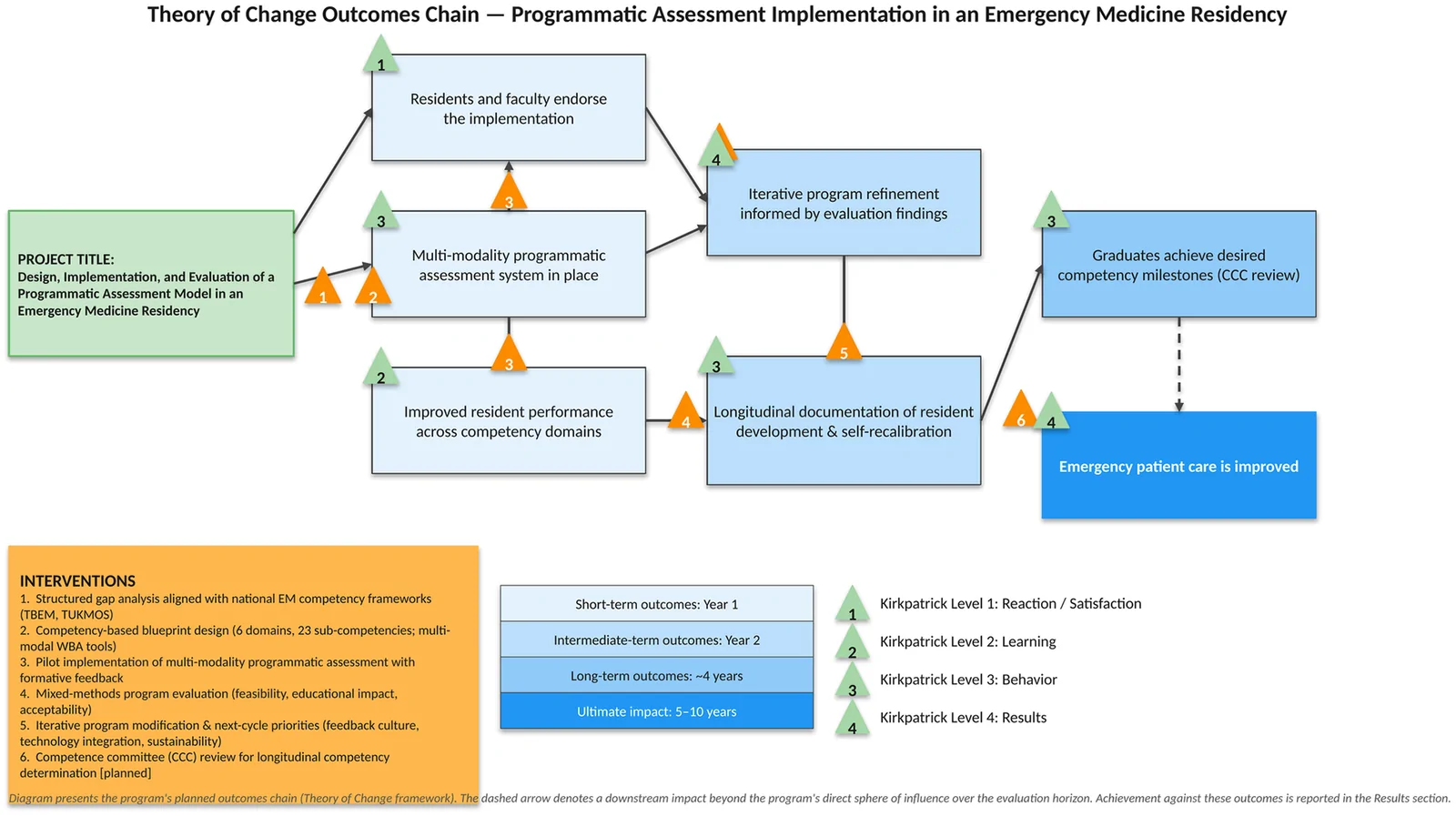
Theory of change outcomes-chain model for the programmatic assessment implementation. Numbered orange triangles mark the six interventions (Phases 1–3); numbered green triangles mark Kirkpatrick training-evaluation levels (L1 = Reaction/Satisfaction; L2 = Learning; L3 = Behavior; L4 = Results). Outcome boxes are colored by time horizon (Year 1 short-term, Year 2 intermediate, ∼4 years long-term, 5–10 years ultimate impact). The dashed arrow denotes downstream impact beyond the program's direct sphere of influence over the evaluation horizon. Framework adapted from WHO technical guidance on evidence-informed Theory of Change (20).

This study used a mixed-methods design to evaluate the introduction of a programmatic assessment model in an emergency medicine (EM) residency program. Program design and evaluation followed an evidence-informed Theory of Change (ToC) framework, which links interventions to their expected short-, intermediate-, and long-term outcomes and ultimate impact, with the underlying assumptions specified at each step (20). We applied the framework's design-phase stages. The problem was defined through a structured gap analysis (Phase 1, below), and expected outcomes were specified across short-term (Year 1), intermediate (Year 2), long-term (∼4 years), and ultimate-impact (5–10 years) horizons. Six interventions with explicit rationales and assumptions were then defined across Phases 1–3, and an outcomes-chain model was constructed to link these elements (Figure 1). Quantitative longitudinal performance data and qualitative resident feedback were collected concurrently during the end-of-pilot evaluation window and integrated descriptively at the interpretation stage via a joint display.

The study was conducted in the EM residency program at Kocaeli University, Türkiye, a four-year competency-based curriculum with five core faculty members. The active resident cohort during the pilot period comprised 20 residents, of whom 19 were eligible for the end-of-pilot evaluation (one resident was unavailable during the survey window). The emergency department (ED) is a high-volume tertiary care unit serving approximately 70,000 patient visits per year. Each shift is staffed by 4–5 residents under the supervision of one core faculty attending. Specialty training in Türkiye is structured under two national frameworks administered by the Ministry of Health and Emergency Medicine Association of Türkiye: the Turkish Board of Emergency Medicine (TBEM) Specialty Training Guide (21) and the Emergency Medicine Core Curriculum of the Medical Specialty Board's Curriculum Development and Standard-Setting System (TUKMOS) (22). Postgraduate medical education in this setting has historically prioritized summative knowledge testing alongside graduated clinical responsibility. Before the pilot, formal assessment in the program consisted primarily of periodic in-training knowledge examinations, with certification resting on the nationally defined end-of-training requirements of a specialty thesis and a final specialty examination. Structured workplace-based assessment was absent: no Mini-CEX, DOPS, multisource feedback, or portfolio was in use, and direct observation of clinical performance was not systematically documented. Day-to-day judgments of resident progress were therefore informal and person-dependent, a pattern later confirmed by the gap analysis (Phase 1).

The study comprised three sequential phases: (1) a preliminary gap analysis, (2) development of an assessment blueprint aligned with national competency frameworks, and (3) pilot implementation and evaluation.

### Phase 1: gap analysis

A structured gap analysis was conducted in August 2024 to examine alignment between existing residency assessment practices and the national EM competency standards in TBEM (21) and TUKMOS (22). Current assessment processes were systematically reviewed against the nationally defined competency domains, milestone expectations, and recommended assessment strategies.

The gap analysis was led by the core EM faculty and included consultation with two national and one international medical education experts. Consultations focused on identifying structural deficiencies, misalignment with competency expectations, areas of under-sampling, and sustainability risks within the existing system.

The analysis identified fragmented assessment practices, lack of triangulation across tools, limited direct observation, person-dependent evaluation processes, and the absence of a centralized system for longitudinal data synthesis. These findings informed priorities for redesign.

### Phase 2: development of the assessment system

Based on the identified gaps, a competency-based assessment blueprint was developed to align the residency program with national standards. Competency domains and sub-competencies were mapped to assessment tools and educational activities. WBAs were structured around entrustable professional activities (EPAs) representing discrete clinical tasks expected of EM residents. Each competency domain was intentionally linked to at least two complementary assessment modalities to support triangulation of performance data.

A structured faculty meeting was convened to refine the blueprint and determine implementation strategies. Core faculty members reviewed the proposed structure and agreed on adaptations suited to the local ED context. The finalized blueprint comprised six competency domains and 23 sub-competencies, supported by a combination of numerically rated tools (whose ratings were aggregated into the longitudinal performance analysis) and narrative-only tools (whose feedback was added to resident portfolios). All modalities were used formatively to generate developmental feedback for residents (Table 1).

**Table 1:** Assessment blueprint of the programmatic assessment model.

Core competencies (*N* = 6)	Medical knowledge and skills	Systems-based practice	Evidence-based medicine and lifelong learning	Communication	Team management and leadership	Professionalism
Sub-competencies (*N* = 23)	1. Triage and emergency stabilization	1. Patient discharge	1. Acquiring updated scientific knowledge	1. Establishing effective communication	1. Demonstrating leadership at every level	1. Professional attitude
	2. Diagnostic skills	2. Pre-hospital emergency services and patient transfer	2. Comparing evidence-based scientific innovations with personal practice and self-evaluation	2. Conducting critical conversations	2. Demonstrating a common attitude and approach	2. Responsibility
	3. Medical treatment and monitoring	3. Disaster and mass-event management	3. Active participation in scientific activities	3. Conflict resolution	3. Creating a solution-oriented team atmosphere	3. Accountability
	4. Procedures	4. Resource management	4. Formulating research questions and designing research		4. Managing situational awareness	
		5. Use of technology				
Assessment tools	Progress examination; Mini-CEX; DOPS; case-based discussion; simulation	360° multisource feedback; Mini-CEX; case-based discussion	360° multisource feedback; mini-PAT (after TBL); seminar evaluations; evidence-appraisal exercise; portfolio reflective writing	360° multisource feedback; Mini-CEX; DOPS; mini-PAT (after TBL); case-based discussion; simulation	360° multisource feedback; mini-PAT (after TBL); simulation	360° multisource feedback; Mini-CEX; DOPS; seminar evaluations; portfolio reflective writing

Sub-competency labels translated from the institutional curriculum aligned with TUKMOS and TBEM frameworks.

### Phase 3: pilot implementation and evaluation

The programmatic assessment model was introduced to faculty and residents in September 2024 through structured orientation sessions outlining its principles, tools, and expectations. Active implementation ran from November 2024 through June 2025, an eight-month pilot period. The five core faculty members (including the program director and department chair) served as both the program-leadership team and the research team for this evaluation. The first author (İÖ) led the project, delivered the orientation sessions, and tracked implementation progress.

Throughout the pilot, the assessment system applied multiple complementary modalities, all used formatively to generate developmental feedback for residents. Numerically rated modalities — WBAs (Mini Clinical Evaluation Exercise [Mini-CEX] and Direct Observation of Procedural Skills [DOPS]), 360-degree multisource feedback, the mini Peer Assessment Tool (mini-PAT), seminar evaluations, and program-wide progress examinations — contributed data points to both the longitudinal performance analysis and ongoing formative feedback. Narrative-only modalities — case-based discussion, simulation, evidence-appraisal exercises, and portfolio reflective writing — contributed feedback that was added to resident portfolios. All ratings and narrative entries were used formatively. No summative-stakes decisions (advancement, ranking, or remediation) were made during the pilot, although comparative information remained visible to residents through the 360-degree feedback reports. As this represented the early implementation phase, no competence committee (CCC) review of portfolios had been conducted at the time of analysis. Data were collected longitudinally using standardized forms. The blueprint specified at least three Mini-CEX and two DOPS per resident across the pilot period (approximately 60 Mini-CEX and 40 DOPS planned across the cohort). Other modalities were not assigned a per-resident frequency target during this first cycle.

Program evaluation comprised one primary and two secondary outcomes. The primary outcome was the feasibility of implementation, operationalized as the volume of completed assessment data points and the extent of participation across competency domains. Secondary outcomes were (i) early educational impact, defined as the domain-level change in resident performance scores between baseline and follow-up rounds, and (ii) acceptability to residents, captured by an anonymous end-of-pilot satisfaction survey of 12 closed-ended items rated on a 5-point Likert scale (1 = strongly disagree to 5 = strongly agree) and four open-ended questions eliciting qualitative feedback. Survey participation was voluntary and anonymous, with no academic consequences.

Iterative modifications to the assessment system were discussed at monthly faculty meetings throughout the pilot, in line with the ToC framework's revision stage (20).

### Quantitative data analysis

Quantitative analysis examined longitudinal changes in cohort-level mean performance across the six competency domains from baseline to follow-up within the eight-month pilot period. The analysis was conducted as a program-evaluation outcome to detect cohort-level signal of educational impact rather than to render individual competency judgments or rank residents for advancement. Individual competency determination is reserved for the CCC. Performance data were derived from two rounds of 360-degree multisource feedback (rated on a 5-point scale, with 3 = expected level) and from two department-administered semiannual progress examinations (100 board-style multiple-choice items covering the full curriculum). Because rater identity and rater stringency varied across the 360-degree feedback rounds and item content varied across the progress examinations, scores were transformed using a within-round norm-referenced approach in which each resident's domain-level mean was divided by the program mean for the same domain in the same round, yielding a ratio in which 1.0 represented the cohort average for that round. Raw scores were not directly comparable across rounds: because the rater pool changed between rounds and the two progress examinations sampled different item content, raw-score change would conflate rater stringency and examination difficulty with change in resident performance. The transformation assumes that the cohort mean provides a stable within-round reference and that rater- and content-related variability affects the cohort approximately uniformly. Accordingly, the reported changes represent shifts in each resident's relative standing within the cohort rather than absolute improvements in competency. Absolute gains shared uniformly by the whole cohort would not be captured by this metric. Longitudinal changes were analyzed using paired-samples t-tests among residents with complete baseline and follow-up data. Magnitude of change was expressed as percentage change (*Δ*%) and effect size as Cohen's d for paired samples. These effect sizes index the magnitude of the cohort-level relative standing shift rather than absolute performance change. Descriptive statistics summarized assessment volume and satisfaction ratings. All analyses were performed in Jamovi (23), with statistical significance set at *p* < .05.

### Qualitative data analysis

Open-ended responses to the four end-of-pilot satisfaction survey questions (strengths, weaknesses, requested changes, and perceived impact on clinical training) were analyzed at the respondent level using an inductive codebook (coding-reliability) approach to thematic analysis (24, 25). Given the small corpus and formative scope, themes were kept at a broad level rather than finely subdivided. Responses were translated from Turkish into English, with translation fidelity checked by a second bilingual reviewer. Two researchers external to the program independently followed the six-phase procedure described by Braun and Clarke — familiarization with the data, generation of initial codes, searching for themes, reviewing themes, defining and naming themes, and producing the report — each producing theme definitions, representative quotations, and a theme-by-respondent presence matrix. Disagreements were resolved through structured consensus reconciliation against merged theme definitions. Consistent with the coding-reliability orientation of this approach, in which themes are consolidated in a shared codebook and coding consistency is treated as an indicator of rigor, inter-coder agreement was quantified using Cohen's *κ* (26). The two coders remained unaware of the core faculty's prior impressions and survey ratings until their analyses were finalized, supporting interpretive independence across data streams. Qualitative themes and survey ratings were integrated descriptively using a joint display (27), pairing each survey construct with the qualitative theme(s) addressing the same content. Given the sample size, no inferential between-stream comparison was undertaken.

## Results

Of 20 active residents, 19 were eligible for the end-of-pilot survey. Seventeen (89.5% of eligible) completed it, and 16 (94% of completers, 84.2% of eligible) provided substantive qualitative feedback suitable for thematic analysis. Sixteen residents had paired baseline and follow-up data from 360-degree evaluations and progress examinations. A total of 3,107 assessment data points were documented during the eight-month pilot, spanning all six competency domains. Two rounds of 360-degree multisource feedback contributed the largest volume (1,466 entries), followed by the mini-PAT (825), seminar evaluations (761), Mini-CEX (35), DOPS (20); two program-wide progress examinations additionally contributed domain-level performance data to the longitudinal analysis. Of the approximately 60 planned Mini-CEXs and 40 DOPS, 35 (58%) and 20 (50%), respectively, were completed across the cohort. Iterative discussions in monthly faculty meetings produced three categories of modification: simplifying overlapping items in WBA forms, refining the timing and feedback-return loop of the mini-PAT, and standardizing narrative-feedback prompts across modalities to reduce documentation burden and support data integration.

**Figure 2:**
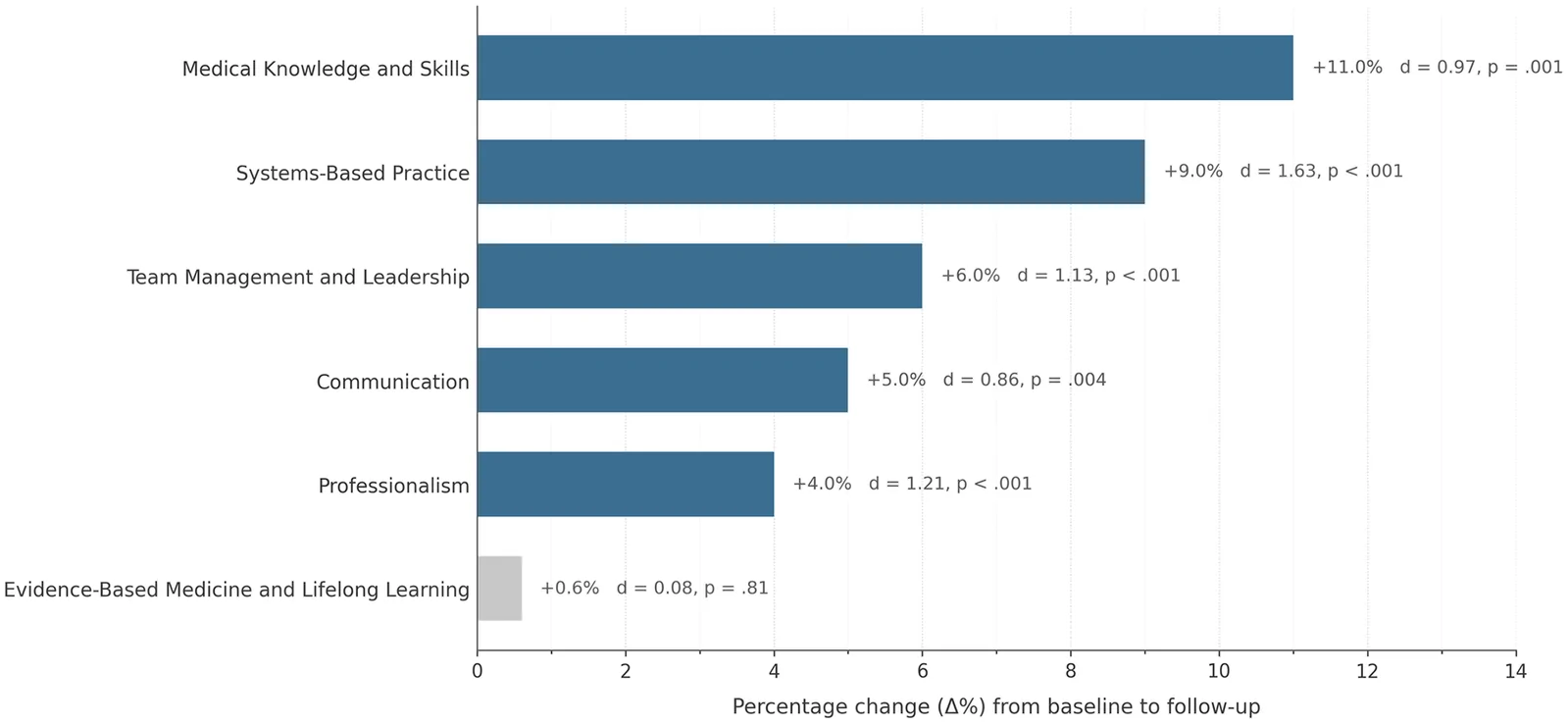
Domain-level cohort progression from baseline to follow-up across the eight-month pilot (*n* = 16 paired). Bars show percentage change in cohort-level domain scores; paired Cohen's d and *p*-value appear next to each bar. Teal bars indicate statistically significant change (*p* < .05); the light-grey bar indicates non-significant change.

Among residents with complete paired performance data (*n* = 16), cohort-level relative standing increased significantly in five of the six competency domains between baseline and follow-up with large effect sizes (Figure 2): Medical Knowledge and Skills (+11.0%, *d* = 0.97, *p* = .001), Systems-Based Practice (+9.0%, *d* = 1.63, *p* < .001), Team Management and Leadership (+6.0%, *d* = 1.13, *p* < .001), Communication (+5.0%, *d* = 0.86, *p* = .004) and Professionalism (+4.0%, *d* = 1.21, *p* < .001). Evidence-Based Medicine and Lifelong Learning did not show significant change (+0.6%, *d* = 0.08, *p* = .81).

Inter-coder agreement on the qualitative themes was strong (Cohen's *κ* = 0.86; per-theme *κ* range 0.50–1.00). The lowest per-theme *κ* corresponded to the bounded-personal-impact theme, where boundary cases between “no impact” and “minimal impact” produced disagreement that was resolved through reconciliation. Acceptability data converged across the end-of-pilot satisfaction survey and qualitative feedback. Mean ratings ranged from 3.4 to 4.1 across the 12 closed-ended items, and six themes were identified across the qualitative responses (Table 2).

**Table 2:** Acceptability findings: end-of-pilot satisfaction survey items (Part A) and qualitative themes (Part B).

Part A. Survey items (*N* = 17).			
#	Item	Mean (SD)	% ≥4
Q1	Forms were easy to complete within the clinical workflow	3.4 (1.2)	41.2%
Q2	Assessment sessions (Mini-CEX/DOPS) did not disrupt my workflow	3.9 (0.9)	58.8%
Q3	The feedback I received was sufficiently detailed	4.1 (0.8)	76.5%
Q4	Faculty feedback was constructive	4.1 (1.1)	70.6%
Q5	The feedback helped me improve my clinical practice	3.8 (0.7)	76.5%
Q6	The collected data were useful for tracking my development	3.6 (1.1)	64.7%
Q7	The program's overall structure supported my learning	3.7 (0.9)	58.8%
Q8	Having many raters made the process fair	3.6 (0.9)	64.7%
Q9	I believe that the scoring is objective	3.5 (1.1)	64.7%
Q10	Overall, I am satisfied with this assessment approach	3.8 (0.4)	76.5%
Q11	I want the program to continue	4.0 (1.0)	82.4%
Q12	The workload is worth the benefit obtained	3.7 (1.0)	58.8%

Part B. Qualitative themes (*n* = 16).		
Theme	*n* (%)	Representative quotation
Valued multisource and workplace-embedded perspective	11 (69%)	“Multisource feedback over time gives a more realistic and objective picture of where I stand than a single exam could.”
Concerns about feedback culture (perceived summative signaling)	11 (69%)	“Even though no decisions are made on these scores, seeing items I scored 3 on flagged in red as below the cohort average makes the feedback feel judgmental rather than developmental.”
Valued longitudinal tracking and self-recalibration	9 (56%)	“It corrects my self-assessment in both directions — areas where I thought I was weak but am not, and areas where I felt strong but actually am not.”
Workload and fit with clinical workflow	5 (31%)	“When on-call shifts are frequent, finding the time to complete the forms and review source material is difficult.”
Concrete design proposals	4 (25%)	“Overlapping items in the forms could be consolidated, and visual elements that imply cross-resident comparison could be reduced.”
Bounded personal impact	4 (25%)	“It is too early in the implementation to judge whether the program is effective for me personally.”

Quotations translated from Turkish and lightly edited for clarity.

Workflow fit produced the lowest item rating (Q1, ease of completing forms within clinical work; mean 3.4, 41.2% endorsing) and was paralleled qualitatively by the dominant practical concern (workload and emergency department workflow burden, 5/16). Ratings on whether observation sessions themselves disrupted workflow were higher (Q2, mean 3.9, 58.8%).

Feedback quality was rated highly (detail, Q3, 4.1, 76.5%; constructiveness, Q4, 4.1, 70.6%) and corresponded qualitatively to a merged developmental theme combining longitudinal tracking, self-recalibration of strengths and weaknesses, and motivation (9/16, 56%). A minority of residents simultaneously reported emotional and psychological costs (3/16), including presentation anxiety and demoralizing score displays.

Rater fairness (Q8, 3.6, 64.7%) and scoring objectivity (Q9, 3.5, 64.7%) sat at moderate endorsement and were mirrored qualitatively by two co-dominant themes (each 11/16, 68.8%): residents valued the external, multisource perspective as a formative-developmental tool while concurrently voicing concerns about the prevailing feedback culture — including rater quality, sampling adequacy, and a perceived summative weight to scoring — with 8 of 16 residents (50%) holding both positions concurrently. The qualitative content distinguished dispositional concerns (careless or biased rating), structural concerns (insufficient rater exposure to the ratee), and explicit reports that certain score-display elements (e.g., red flagging of below-average items in the 360-degree feedback) made the experience feel summative-evaluative for some residents, even though no summative-stakes decisions (advancement, ranking, or remediation) were made on the basis of these scores during the pilot.

Program structure (Q7, 3.7, 58.8%), overall satisfaction (Q10, 3.8, 76.5%), continuation (Q11, 4.0, 82.4%) and cost–benefit (Q12, 3.7, 58.8%) were generally positive. Four residents (4/16, 25%) reported a bounded personal impact attributable to timing, prematurity of the pilot, dispositional neutrality, or limits on changing operational flow.

Four residents volunteered concrete design proposals: reducing overlapping items in the assessment forms, phasing the program in with incoming cohorts rather than residents already mid-training in the traditional assessment system, and prioritizing immediate face-to-face bedside feedback after observed encounters alongside the written scores. A further suggestion concerned redesigning score-display elements that residents perceived as implying cross-resident comparison. This proposal is reflected in the feedback-culture theme above (Theme 2).

Across constructs, the integration was largely confirmatory. The rater-fairness and scoring-objectivity items (Q8, Q9) sat at moderate endorsement and were paralleled qualitatively by the dual formative-developmental and perceived summative-evaluative readings — an instance of expansion in the joint-display sense (27).

## Discussion

We designed and piloted a programmatic assessment (PA) model in an emergency medicine residency at a high-volume tertiary care center, working with a small core faculty and a comparatively large resident cohort. The system was developed following a structured gap analysis informed by national competency frameworks and consultation with national and international medical education experts, and was implemented over an eight-month pilot. Although the educational impact of a PA system is most fully evaluated over a longer time horizon, this initial cycle was sufficient to document a competency development signal across most domains and to characterize the system's acceptability among learners. The findings position our experience within an international PA literature dominated by Canadian and United States programs (5) and provide an early account from a setting in which PA is being introduced against a historically summative assessment culture. Our systematic review of PA in postgraduate medical education confirmed how thin and concentrated this evidence base is: of 20 empirical studies, only four measured competency-related outcomes, all with observational single-cohort designs, and 80% originated from Canada or the United States, with lower-resource settings entirely unrepresented (5).

At the program level, implementation was feasible: 3,107 data points were recorded across multiple low-stakes modalities, all six competency domains were sampled, and 16 residents had complete paired performance data. Cohort-level relative performance moved in five of six domains over the eight-month pilot. These findings are broadly aligned with prior PA implementations once a system is operationalized (7, 8, 28) and with observational reports of measurable post-implementation change (14–16). As in those reports, our impact data are derived from system-internal metrics in a single-cohort pre–post design without an external comparator (7, 8, 15), and the analysis reflects cohort-level signal rather than absolute performance change. Because the same assessment system both delivered the intervention and generated the outcome data, the observed changes should be read as evidence that the system can detect a developmental signal, not as independent confirmation of educational effectiveness. Independent external outcome measures — national board examination results, faculty global ratings collected outside the system, milestone determinations, competence-committee decisions, and patient-level outcomes — were not available during this first cycle to corroborate the signal. In this respect our design mirrors the field at large: in our systematic review of PA in postgraduate medical education, all four studies reporting competency outcomes relied on system-internal metrics, and none measured clinical performance or patient-care outcomes (5). The single domain without significant change — Evidence-Based Medicine and Lifelong Learning — was sampled in our blueprint primarily through educational activity modalities rather than through clinically embedded observation. A modality–construct mismatch is plausible: standardized performance assessments have been shown to detect competency deficits that workplace-based global ratings can miss (10). Future iterations will pair the existing modalities with clinically embedded EBM observations.

A specific feature of our implementation is the comparatively low volume of documented WBAs — 35 Mini-CEX and 20 DOPS entries across the eight-month pilot — alongside extensive informal bedside observation and verbal feedback, which occur routinely in this setting. Only 58% of planned Mini-CEX and 50% of planned DOPS encounters were completed. This shortfall is itself a substantive feasibility finding: the blueprint's direct-observation targets were not attainable under current staffing and workflow conditions, and the modality that provides the most direct evidence of clinical performance was consequently under-sampled relative to plan. Next-cycle targets will therefore need to be paired with workflow changes that lower the cost of documentation, or revised toward volumes demonstrably achievable in this setting. The pattern reflects a feedback-culture norm in which observation and corrective conversation are familiar, while structured documentation is harder to integrate into a high-volume ED workflow staffed by a small core faculty. Variability in the proportion of clinical observation that is formally documented has been described in PA implementations even in resource-rich settings (7, 8), and the under-sampling of direct observation has been identified as a recurring implementation barrier in emergency medicine specifically (7, 11). Our systematic review found feedback-documentation barriers reported consistently across settings and specialties, suggesting that this is an inherent feature of PA implementation rather than a context-specific difficulty (5). The implication is that the gap between informal observation and formal documentation is a documentation problem rather than an observation problem, and that closing the gap is more likely to come from tools that make capture frictionless (29) than from adding stand-alone assessment events.

The acceptability findings extend prior work in two related directions. First, two qualitative themes were co-dominant (each 11/16) and represented opposing readings of the same multisource system: a formative-developmental reading that valued external perspectives and longitudinal feedback, and a perceived summative-evaluative reading expressed as feedback culture concerns. The latter clustered around rater quality, sampling adequacy, and the way certain visualization features in the 360-degree feedback were experienced by some residents as judgmental rather than developmental, even though no individual-level summative comparisons or decisions were made during the pilot. Eight of 16 residents (50%) held both readings simultaneously. The pattern parallels findings from emergency medicine and other specialties — faculty culture and attending perceptions of WBA (13), assessor failure-to-fail in EM (6), and resident-archetype effects on competency-committee functioning across specialties (17) — together with avoidance behaviors under uncertainty in internal medicine (18) and the checkpoint-fatigue construct described internationally (19). Second, workload was the dominant practical concern (5/16) and aligned with onerousness signals across PA implementations (7, 8, 14, 28). Notably, despite the concerns described above, 82.4% of residents endorsed continuing the program, suggesting they viewed the system as one to refine rather than reject. Several contextual features of our setting likely contribute to both where our findings converge with and where they diverge from the published evidence. First, a small core faculty supervising a comparatively large resident cohort accounts in part for the workload pattern reported across PA implementations (7, 8, 28). Second, a clinical feedback culture in which bedside verbal correction is more familiar than structured documentation underlies the gap we describe between extensive informal observation and lower formal documentation. Third, a national assessment tradition historically weighted toward summative testing (21, 22) plausibly amplifies the perception of summative signaling among residents encountering a sustained low-stakes feedback structure for the first time. The stakes-as-dichotomy construct described by Schut et al. (19), however, is structural across PA implementations regardless of national assessment culture, so the Turkish summative tradition is best understood as amplifying rather than producing the gap. Consistent with this reading, our systematic review found misalignment between intended and perceived assessment stakes reported consistently across settings, alongside variable stakeholder engagement and substantial resource requirements (5).

These findings define a concrete agenda for subsequent cycles. The single most important priority is strengthening feedback culture and resident familiarity with PA, so that the formative-developmental intent of the system is reliably perceived as such. This work includes structured PA-orientation sessions for incoming residents and explicit communication that the system is formative. Rather than attempting to standardize assessors through calibration alone, faculty development will prioritize the quality and learning value of feedback conversations. Individual judgments are inherently subjective, and programmatic assessment treats that subjectivity as informative rather than as error to be eliminated, provided many low-stakes data points are aggregated (2, 30). Helping residents understand this property of the system is part of the same agenda. Establishing a formal competence committee is a parallel structural priority, since committee-based synthesis both completes the programmatic-assessment cycle and shapes how learners perceive the stakes of the system (17). The sustainability of implementation will be examined in parallel, including faculty time, data management burden, and continuity of engagement (8, 28). Technology integration will support several specific aims. A tablet- or mobile-based platform will be used to optimize the frequency and quality of direct observations (29). Streamlined interfaces and templated prompts will reduce faculty workload in synthesizing narrative feedback. An integrated dashboard-ePortfolio will consolidate numeric and narrative data into a single resident- and faculty-facing view, supporting both ongoing developmental conversations and forthcoming CCC review (28). Finally, AI-assisted summarization of narrative feedback will be piloted to reduce the cognitive burden on residents and faculty (31). Visualization features experienced as summative will be redesigned, and the blueprint will be extended to include clinically embedded EBM observations.

## Limitations

This study has several limitations. It is a single-center pilot with 17 survey respondents and 16 residents in the qualitative analysis. Findings are descriptive rather than generalizable. Generalization beyond our setting should attend to the structural conditions that produced these findings: shift-based EDs with small core faculty operating under a national framework that has historically emphasized summative testing. The performance-change analysis used a single-cohort pre–post design and shares the circularity caveat raised in the broader PA literature, in which the system both generates the data and defines the outcome (5, 7, 8, 15). Without a concurrent comparison group or an interrupted time-series design, the observed changes cannot be attributed to the assessment program itself. Natural training progression and maturation over the eight-month interval, practice and familiarity effects, and regression to the mean are plausible alternative explanations for some or all of the observed change. For the single domain without significant change (Evidence-Based Medicine and Lifelong Learning), regression to the mean and insufficient curricular dose during this short window are plausible alternative explanations that cannot be adjudicated from the present data. Baseline score distributions were not systematically examined for ceiling effects in this evaluation, so a ceiling effect can be neither supported nor excluded. The norm-referenced transformation supports cohort-level relative-standing inference rather than absolute performance change. This analysis served program evaluation rather than individual competency determination, while acknowledging the principled critique of norm-referencing within CBME (32). Several blueprint modalities (case-based discussion, simulation, evidence-appraisal exercises, and portfolio reflective writing) generated narrative feedback that was added to resident portfolios, but was not represented in the quantitative analysis, and no competence committee review of portfolios had yet been conducted at this stage. Faculty perspectives were not captured in the qualitative dataset because the same five core faculty members served both as program leadership and as members of the research team, and eliciting candid faculty narrative feedback under those conditions would have introduced a substantial conflict of interest. Faculty perspectives will instead be captured in subsequent cycles by independent qualitative researchers. Qualitative resident analysis was performed on translated responses. Subtle Turkish-language nuance may have been lost despite translation-fidelity checks. Finally, longer-term educational outcomes and patient- or healthcare-system-level outcomes — the broader downstream impact that SQUIRE-EDU encourages educators to consider — were outside the scope of this report (33) and remain explicit gaps the program will address.

## Conclusion

Implementation of a programmatic assessment model in this Turkish emergency medicine residency was feasible and broadly acceptable, and was accompanied by measurable gains in cohort-level relative performance in five of six competency domains, although the uncontrolled pre–post design precludes attributing these changes to the program itself. Two coexisting tensions warrant continued attention: residents simultaneously valued the multisource perspective and doubted rater objectivity, and broadly endorsed the program while reporting a burden of form completion amid the busy clinical duties shouldered by both residents and faculty. The most concrete next-cycle priority is reducing documentation burden while strengthening rater exposure and the learning value of feedback. By contributing data from a setting currently absent from the international PA literature, this evaluation extends the evidence base for the portability of programmatic assessment across different workforce structures and assessment cultures.

## Data Availability

The raw data supporting the conclusions of this article will be made available by the authors, without undue reservation.
